# SparNet: A Convolutional Neural Network for EEG Space-Frequency Feature Learning and Depression Discrimination

**DOI:** 10.3389/fninf.2022.914823

**Published:** 2022-06-02

**Authors:** Xin Deng, Xufeng Fan, Xiangwei Lv, Kaiwei Sun

**Affiliations:** Key Laboratory of Data Engineering and Visual Computing, College of Computer Science and Technology, Chongqing University of Posts and Telecommunication, Chongqing, China

**Keywords:** SENet, SparNet, space-frequency domain characteristics, depression, EEG

## Abstract

Depression affects many people around the world today and is considered a global problem. Electroencephalogram (EEG) measurement is an appropriate way to understand the underlying mechanisms of major depressive disorder (MDD) to distinguish depression from normal control. With the development of deep learning methods, many researchers have adopted deep learning models to improve the classification accuracy of depression recognition. However, there are few studies on designing convolution filters for spatial and frequency domain feature learning in different brain regions. In this study, SparNet, a convolutional neural network composed of five parallel convolutional filters and the SENet, is proposed to learn EEG space-frequency domain characteristics and distinguish between depressive and normal control. The model is trained and tested by the cross-validation method of subject division. The results show that SparNet achieves a sensitivity of 95.07%, a specificity of 93.66%, and an accuracy of 94.37% in classification. Therefore, our results can conclude that the proposed SparNet model is effective in detecting depression using EEG signals. It also indicates that the combination of spatial information and frequency domain information is an effective way to identify patients with depression.

## 1. Introduction

Major depressive disorder (MDD, also known as unipolar depression) is a physical disease of the brain, also known as a mental health disorder. It mainly affects the process of thought, behavior, and mood, and also can lead to the loss of interest and energy, interpersonal relationships, and job performance. According to the statistics of the World Health Organization, more than 300 million people in the world suffer from depression, and about 800,000 people die of depression every year (Belmaker and Agam, 2008; Olesen et al., 2012; Whiteford et al., 2013). Early and accurate diagnosis of depression is crucial for patients who need timely clinical treatment.

Most of the previous diagnoses of depression are based on the questionnaires as a judgment and screening tool. One of the major drawbacks of this method is that it requires experienced doctors. Therefore, finding a suitable and effective way to detect depression is an emerging research area. Currently, various physiological measurement tools are developing rapidly, such as functional magnetic resonance imaging (fMRI), electroencephalogram (EEG), and positron emission tomography (PET). Many studies attempt to measure psychological data and develop auxiliary diagnostic methods in clinical practice (Van Der Stelt and Belger, 2007; Michel and Murray, 2012; Olbrich and Arns, 2013; Kerestes et al., 2014; de la Salle et al., 2016). The quantitative measurement of EEG signals is a neuroimaging technique with obvious practical advantages because it does not involve invasive manipulation, and it is easy to manage, well-tolerated and relatively inexpensive. In addition, the prevalence and persistence of depressive symptoms make scalp recording EEG an appropriate method to understand the underlying mechanisms of depression.

Most of the existing deep learning methods take the original data of EEG signals or transform them into frequency-domain signals as input, thus losing the spatial features between multiple brain regions and the whole brain. Liao et al. (2017) and Jiang et al. (2021) showed the effectiveness of spatial information in distinguishing depression. Considering that the irregular EEG network is one of the possible physiological symptoms of depression, the EEG activity has spatial characteristics originating from different brain regions, so the spatial EEG characteristics extracted from different brain regions can be used to identify depression. Cai et al. (2018) also found that the features in the frequency domain were more likely to distinguish patients than those in the time domain. By fusing these two features of comprehensive mixed information, it is expected to achieve richer and more accurate identification of depression. Although it is possible to explore the spatial information of the brain based on the internal structure of neural networks, there are seldom studies that have explored the spatial information of the various brain regions in depression based on the whole brain structure from EEG signals. The purpose of this study is to combine the spatial information and the frequency domain information of each brain region, and integrates them with deep learning.

The main contributions of this study are as follows. First, phase space reconstruction is used to denoise the EEG signals in the time domain and smooth the feature in the frequency domain. Second, a new model called SparNet is proposed to capture more specific information about depression in this study. It is a parallel convolutional network used to extract the features of different brain regions, and the attention mechanism module is added to the network. Third, the channels for each brain region are selected to explore the local spatial-frequency domain features. The frequency domain and the spatial features of each brain region are combined by the multi-layer parallel convolutional filter. By adding the attention mechanism, our deep learning model can assign the weights to different channels in the local brain region and also to different contribution degrees in the global brain region.

## 2. Related Study

All approaches to depression identification fall into two broad categories: those based on the manual features and those based on the raw data. Hosseinifard et al. (2013) used a large EEG recording dataset of 90 subjects (45 normal subjects and 45 depressed subjects) and found that in non-linear features, the correlation dimension is a powerful feature for analyzing EEG signals and identifying the depressed and the non-depressed subjects. Cai et al. (2018) through three-electrode channel acquisition, found that features were mainly concentrated in the frequency domain, and achieved the best accuracy of 79.27% with KNN. Their recent study, Cai et al. (2020b) compared KNN, DT, and SVM on the same data set. Their KNN model achieved the highest accuracy of 89.98%. Liao et al. (2017) proposed a nuclear feature filtering group common space Mode (KEFB-CSP) based on the scalp EEG signals. The signals were decomposed into each frequency band and then the spatial features were extracted by the CSP algorithm. Mumtaz et al. (2017) conducted the time-frequency decomposition of an EEG data and constructed EEG data matrix. Compared with other time-frequency methods such as STFT and EMD, the wavelet analysis has the highest classification accuracy of 87.5%. Mahato and Paul (2020) found that the average theta asymmetry of normal people was higher than that of patients with depressive people. In SVM, the classification accuracy of alpha2 and theta asymmetric combination is 88.33%. Peng et al. (2019) collected 128 electrodes of the subjects, and the research results showed that depression would affect the brain activity of almost the entire cerebral cortex, and the accuracy of 92.73% was achieved by using SVM and full frequency band features. Sun et al. (2020a) found that there were far more functional connections within hemispheres than between hemispheres. High frequency parietal occipital lobe plays an important role in depression recognition. They Sun et al. (2020b) achieved the highest classification accuracy of 82.31% by using the ReliefF feature selection method and LR classifier on the same data set. They further indicated that the functional connection feature plays an important role in depression recognition. Jiang et al. (2021) proposed an effective EEG based spatial classification detection method for depression, task-related common spatial pattern (TCSP), which significantly improved the accuracy of depression classification by using spatial information.

Although feature extraction and machine learning can effectively identify patients with depression, manual feature extraction and selection are required, which is time-consuming and laborious. There are many studies that use raw EEG data or pre-processed data as model input. Zhang et al. (2020) extracted the temporal and spatial characteristics of EEG signals by 1DCNN and added the population attention mechanism. They suggested that the combination of EEG signals and demographic factors could be better for patients with depression. Fan et al. (2020) combined CNN and LSTM to better extract time and space information. Ke et al. (2020) designed a lightweight CNN model for the online identification of patients with depression. Wan et al. (2020) proposed a convolutional neural network HybridEEGNet composed of two parallel lines for learning synchronization and regional EEG features. Seal et al. (2021) found that the right extreme value of the subjects with depression was significant, while the left extreme value of normal subjects was significant. Sharma et al. (2021) proposed a computer-aided (CAD) hybrid neural network based on EEG, that used CNN for time learning and LSTM architecture for sequence learning.

In the detection of depression, the spatial and frequency domains are two important factors, but there are no suitable neural networks to combine them together. Therefore, we propose the SpatNet neural network to combine the two features to improve the detection of depression.

## 3. Materials and Methods

### 3.1. Participants

The dataset used in this study is from Lanzhou University. The dataset (Cai et al., 2020a) mainly includes data from patients with depression and the normal control group. Prior to the experiment, all participants signed the written informed consent. The consent and study design were approved by the Local Biomedical Research Ethics Committee of Lanzhou University Second Hospital in accordance with the World Medical Association rules. There were 48 participants, including 24 patients with depression (13 men and 11 women; 16–56 years old) and 24 healthy controls (17 men and 7 women; 18–55 years old). All patients with depression underwent a structured MINI interview, which met the diagnostic criteria for depression in the DSM-IV-based Diagnostic and Statistical Manual of Mental Disorders (DSM). Patients with MDD were selected based on their PHQ-9 (Kroenke and Spitzer, 2002), GAD-7 (Spitzer et al., 2006), and PSQI scores. Participants should be between 18 and 55 years old and have primary or higher education. For depression, inclusion criteria were MINI meeting the diagnostic criteria, patient health Questionnaire (PHQ-9) score of 5 or greater, and no psychotropic medication in the past 2 weeks.

### 3.2. Data Acquisition and Preprocessing

Subjects were asked to stay awake and still, and to reduce head and body movements and eye movements to reduce EMG and EOG, respectively, which record a 5-min resting state of closed eyes. EEG signal acquisition equipment is 128-channel HydroCel Geodesic Sensor Net. The sampling rate is 250 Hz. The reference electrode is Cz. The skin impedance of each electrode channel is kept below 70 kΩ.

**Figure 1:**
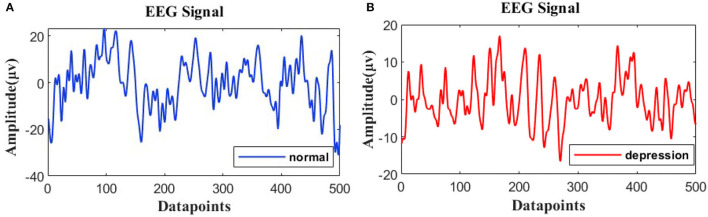
Electroencephalogram (EEG) signals from **(A)** normal and **(B)** depressed subjects.

The resting EEG signals are further processed using MATLAB 2021b. In the first step, the infinite impulse response digital filter IIR is used to perform 1–40 Hz band-pass filtering on the signal, and the order of the filter is set as 6. The filter can eliminate the “baseline drift” caused by low frequency noise and electrical interference from the 50 Hz-line noise. In the second step, independent principal component analysis is used to remove the EOG and EMG. Meanwhile, the integrity of channel recording signals is checked. If the invalid channels are detected, spherical interpolation is used for interpolation. The EEG signals of depressed patients and normal people are shown in Figure 1. In the third step, the processed time-domain signal is decomposed and reconstructed to remove the noise. A phase space reconstruction of a time-domain signal is decomposed into three signals, and then the new signal is reconstructed through the least square interpolation. The fourth step is to keep the same sample size between the subjects. The sliding window with the 2s non-overlap method is adopted for sectioning (Siuly et al., 2015), and the sample size of each subject is 148*2s. In the process of beginning and ending the experiment, the interference of brain electricity would be relatively large, so we discarded the first sample and the last sample.

### 3.3. The Time-Domain Denoising

#### 3.3.1. Phase Space Reconstruction

Compared with normal EEG signals (Knott et al., 2001; Puthankattil and Joseph, 2012; Sharma et al., 2018), inhibited EEG signals have stronger stability and lower complexity. Hence, the phase space reconstruction is used to better analyze the complexity and non-stationary behavior of normal and depressed EEG signals in two-dimensional space, and the unstable noises are removed. In Sharma and Pachori (2015), Bhattacharyya and Pachori (2017), the two-dimensional diagrams of EEG signals have been used for seizure detection. The phase space reconstruction was first proposed in 1980. There are mainly two methods for phase space reconstruction: the derivative reconstruction method and the coordinate delay reconstruction method. The data set is collected in the resting state, so the signal is generally stable. The current signal feature can be predicted by the previous signal feature and the following feature. Based on this characteristic, the coordinate delay reconstruction method is finally adopted. The generation of the PSR requires the determination of delay time t and embedding dimension d, which can be obtained by mutual information (MI) (Roulston, 1999; Bradley and Kantz, 2015) and false nearest Neighbor (FNN) (Bradley and Kantz, 2015), respectively. Supposing the time series is *x*(*i*): *i* = 1, 2, …, *n*, the d dimensional phase space vector is constructed by different delay time t of one-dimensional time series *x*(*i*), as shown in Equation (1).

(1) $$\begin{array}{r} y\left(i\right)=\left(x\left(i\right),\ldots ,x\left(i+\left(d-1\right)t\right)\right),1\leq i\leq n-\left(d-1\right)t \end{array}$$

The initial application of the PRS is in the chaotic time series. According to Takens' embedding theorem (Stark et al., 1997; Muldoon et al., 1998; Kukavica and Robinson, 2004), we can reconstruct a phase space from one-dimensional chaotic time series that is the same as that of the prime motorial system in the sense of topology. According to this principle, the EEG signals as the general time series can reconstruct a phase space topologically identical to the original signal by determining the delay time t and d dimensions of the phase space. Since the EEG signal is collected by the subject in the resting state with eyes closed, the brain activity is relatively stable, and the characteristics of the current signal can be determined by the characteristics of the front and rear signals. If there are interference noises at this time, the original signal will have a mutation in the waveform. We can determine and correct the reconstructed phase space.

#### 3.3.2. Least Square Fitting

After the original EEG signal is reconstructed in the phase space, the reconstruction is selected as a three-dimensional phase space vector. Then, the linear least square method by Kiers (1997) is used to fit each point inside, because the EEG signal in the resting state would not have mutations. Thus, the current signal point could be fitted according to the characteristics of the signals before and after. Given n points (*x*_*i*_, *y*_*i*_), *i* = 1, 2, 3, …, *n*. *x*_*i*_ is not the same, as shown in Equation (2).

(2) $$\begin{array}{r} f\left(x_{i}\right)=a_{1}r_{1}\left(x_{i}\right)+a_{2}r_{2}\left(x_{i}\right)+\cdots +a_{m}r_{m}\left(x_{i}\right) \end{array}$$

*f*(*x*) is closest to all the data points. We assume that the current data point is *i* (unknown) and all known points before and after are taken to determine the fitting function. Where, the steps to determine the coefficient *a*_*k*_ are as follows: First, the error function of the fitting curve and the original curve is Equation (3).

(3) $$\begin{array}{r} J\left(a_{1},a_{2},\ldots ,a_{m}\right)=\overset{n}{\underset{i=1}{\sum }}\delta^{2}=\overset{n}{\underset{i=1}{\sum }}{\left[f\left(x_{i}-y_{i}\right)\right]}^{2} \end{array}$$

For *a*_1_, *a*_2_, …, *a*_*m*_ minimizes *J*, use the necessary extreme conditions: ∂*J*/∂*a*_*k*_ = 0 (*k* = 1, …, *m*) to get the linear Equation (4).

(4) $$\begin{array}{r} \overset{n}{\underset{i=1}{\sum }}r_{j}\left(x_{i}\right)\left[\overset{m}{\underset{k=1}{\sum }}a_{k}r_{k}\left(x_{i}\right)-y_{i}\right]=0,\left(j=1,\ldots ,m\right) \end{array}$$

make *R*= $\left(\begin{array}{ccc} r_{1}\left(x_{1}\right) & \cdots & r_{m}\left(x_{1}\right)\\ \vdots & \ddots & \vdots \\ r_{1}\left(x_{n}\right) & \cdots & r_{m}\left(x_{n}\right) \end{array}\right)$, $A={\left[a_{1},\ldots ,a_{m}\right]}^{T}$, $Y={\left[y_{1},\ldots ,y_{n}\right]}^{T}$, so we can rewrite this equation as Equation (5).

(5) $$\begin{array}{r} R^{T}RA=R^{T}Y \end{array}$$

Thus, when the equation satisfies *r*_1_(*x*), …, *r*_*m*_(*x*) linearly independent, *R* column full rank, *R*^*T*^*R* invertible, there is a unique solution Equation (6).

(6) $$\begin{array}{r} A={\left(R^{T}R\right)}^{-1}R^{T}Y \end{array}$$

**Figure 2:**
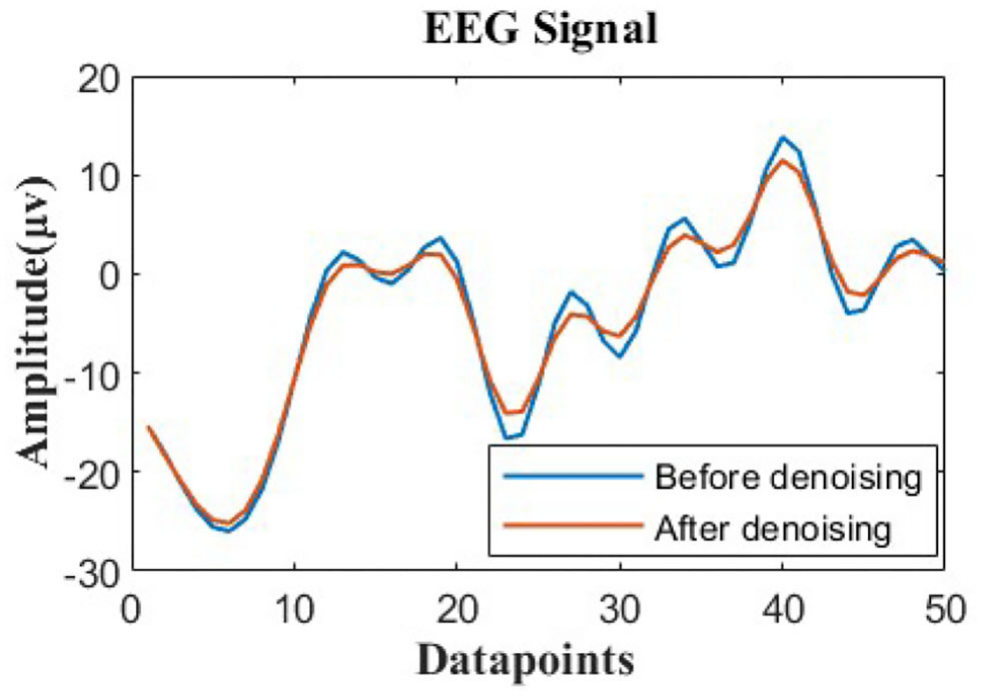
Phase space reconstruction and the least square method were used to detect EEG signals before and after denoising in patients with depression.

For the selection of function *r*_*k*_(*x*), we use the polynomial curve to better fit the EEG signal. Figure 2 shows the comparison before and after denoising EEG signals of patients with depression by using phase space reconstruction and the least square method.

### 3.4. Characteristics of Feature Smoothing

Pham et al. (2015) proposed the importance of feature smoothing for emotional EEG classification. The denoised EEG signal is converted into the frequency domain signal by the fast Fourier transform. The frequency domain signal is used as the input of the neural network. Each sample is segmented according to the time sliding window. Therefore, the transformed frequency domain signals are also smoothed according to 148 time points (each sample size). The EEG signals are collected by the subjects with their eyes closed, so the changes in the EEG signals in the frequency domain are not particularly obvious. Equations (7) and (8) are used to calculate the difference between the signals at each time point and the mean value of the signals.

(7) $$\begin{array}{r} \delta_{j}^{i}=|y_{j}^{i}-E{\left(Y\right)}^{i}|,1\leq i\leq 40,1\leq j\leq 148 \end{array}$$

(8) $$\begin{array}{r} E{\left(Y\right)}^{i}=\frac{1}{J}\overset{J}{\underset{j=1}{\sum }}y_{j}^{i} \end{array}$$

Where *Y* represents the amplitude, *i* represents the frequency, and *j* represents the time point. *E*(*Y*) is the mean of a sample of a subject at frequency *i*. $\delta_{j}^{i}$ is the error between each sample point and the mean.

(9) $$\begin{array}{r} \delta_{j}^{i}-3\sqrt{\frac{1}{J}\overset{J}{\underset{j=1}{\sum }}{\left(y_{j}^{i}-E{\left(Y\right)}^{i}\right)}^{2}}>0 \end{array}$$

Mark $y_{j}^{i}$ as an outlier if the value of $y_{j}^{i}$ differs from the mean by more than three standard deviations as shown in Equation (9). After the outliers are detected, the $y_{j}^{i}$ sample points marked before are smoothed by the method of before-and-after mean interpolation. In this way, the influence of abnormal features can be further cleaned up, while maintaining the overall trend of frequency domain features. At the same time, it can reduce the risk of over-fitting during the training of the neural network.

### 3.5. Attention Module

The attention mechanism is a special structure embedded in a machine learning model, which is used to automatically learn and calculate the contribution of input data to output data. The attention mechanism is a signal processing mechanism discovered by some scientists in the study of human vision. Some practitioners in the field of artificial intelligence have introduced this mechanism into some models. At present, the attention mechanism has become one of the most widely used “components” in the field of deep learning, especially in the field of natural language processing. The classic ones are BERT, Transformer (Devlin et al., 2018; Wang et al., 2019), and other models or structures that are highly exposed in the past 2 years. In this article, we adopt the SENet (Squeeze-and-Excitation Networks) (Hu et al., 2018) module incorporated with the channel attention mechanism.

**Figure 3:**
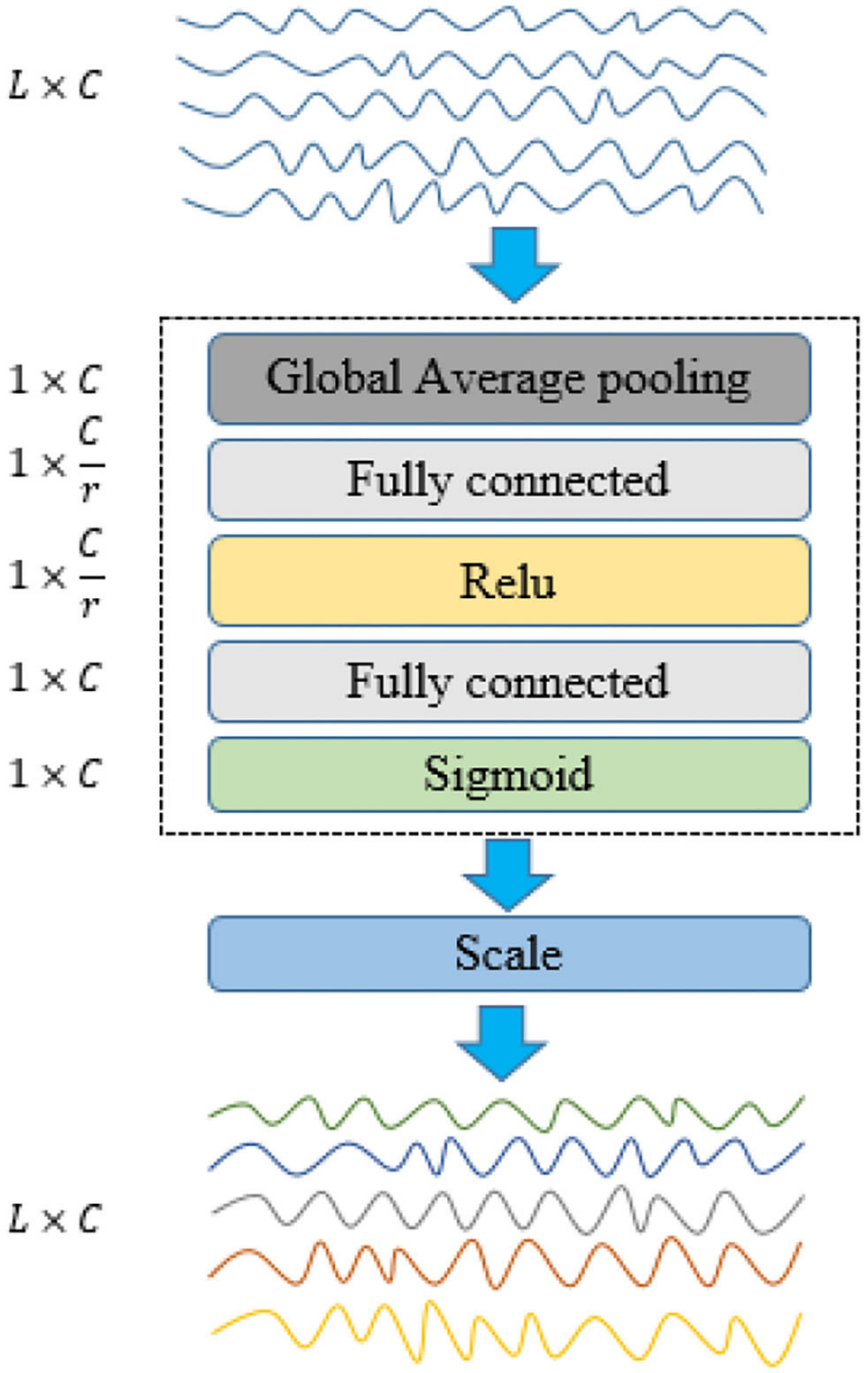
Structure diagram of SENet network applied to one dimensional EEG signal. The weight of each channel of EEG signal before processing is equal, so the color of the channel is the same. The different channels after processing were assigned different weights, with different colors representing this change.

In our proposed network model, the EEG signals are converted into frequency domain features and then used as the input of the SENet module. The SENet module is originally used to process the two-dimensional images, in this article, we use it in one-dimensional signal processing. It can mainly use the global information to selectively emphasize the information features and suppress the less useful features by assigning different weight values to each channel. This is a combinatorial function of five consecutive operations: channel global average pooling (Lin et al., 2013), complete connection layer, Relu, complete connection layer, and finally Sigmoid. The sigmoid activation plays an important role as the channel weights that adapt the input specific descriptors. Due to the fully connected layer and pooling layer, the number of parameters and the computation load increased slightly. The unique structure of this extrusion and excitation network, shown in Figure 3, can be used with any standard architecture.

#### 3.5.1. Squeeze

SENet implements compression operations through the global average pooling to generate channel statistics. Where *Z* ∈ *R*^*C*^, and the *k*^*th*^ element *z*_*k*_ of *Z* is calculated by Equation (10).

(10) $$\begin{array}{r} z_{k}=F_{sq}\left(u_{k}\right)=\frac{1}{L}\overset{L}{\underset{i=1}{\sum }}u_{k}\left(i\right),k=1,2,\cdots \;,C \end{array}$$

where *F*_*sq*_(·) is the compression operation, and *u*_*k*_ is the feature on the *k*^*t*^*h* channel. *C* is the total number of channels.

#### 3.5.2. Excitation

The excitation operations help to capture channel dependencies and greatly reduce the number of parameters and calculations. The excitation part is mainly composed of two fully connected layers and two activation functions, which can be written as Equation (11).

(11) $$\begin{array}{r} S=F_{ex}\left(Z,W\right)=\sigma \left(\gamma \left(Z,W\right)\right)=\sigma \left(W_{2}\delta \left(W_{1}Z\right)\right) \end{array}$$

where *S* = *s*_1_, *s*_2_, …, *s*_*C*_,$s_{k}\in R^{L}\left(k=1,2,\ldots ,L\right)$. *F*_*ex*_(·) is the excitation operation. $W_{1}\in R^{\frac{C}{r}\times C}$, $W_{2}\in R^{C\times \frac{C}{r}}$, *W*_1_, and *W*_2_ are the weights of the two fully connected layers used for dimension reduction and dimension enhancement. *Z* is the fully connected input after global average pooling. *r* is a hyperparametric ratio, which can change the capacity and calculation cost. δ(*x*) is the activation function Relu used to prevent the gradient from disappearing (Gu, 2017). $\sigma \left(x\right)=\frac{1}{\left(1+e^{-x}\right)}$ is a sigmoid function. Equation (12) is used to calculate the final output $\tilde{x_{k}}\left(k=1,2,\cdots \;,C\right)$. The output is obtained by multiplying the input channels by their respective weights.

(12) $$\begin{array}{r} \tilde{x_{k}}=F_{scale}\left(u_{k},s_{k}\right)=u_{k}\cdot s_{k} \end{array}$$

where $\tilde{x_{k}}\in R^{L}$ refers to the multiplication above the channel. *s*_*k*_ is the processed channel weight. *u*_*k*_ is the original eigenvector.

## 4. Proposed Deep Learning Scheme

Convolutional Neural Network is a special type of neural network which is widely used in image processing and classification tasks. It is a state-of-the-art deep learning method consisting of many stacked convolutional layers. The network consists of a convolution layer, pool layer, and final complete connection layer. The EEG signals are one-dimensional time series signals. After converting them into frequency domain signals, multi-channel one-dimensional frequency domain signals are input. Therefore, 1DCNN is used in the convolutional neural network. The features in the frequency domain can be fully combined with the spatial information between channels.

### 4.1. Convolution Layer

At the convolution layer, 1DCNN carries out the convolution operation on the local area of the input signal to generate the corresponding one-dimensional feature map. Different convolution kernels extract different features from the input signal respectively. Each convolution kernel detects the specific features of all positions on the input feature graph to achieve the weight allocation on the same input feature graph. The characteristics of the local connectivity and weight sharing effectively can reduce the complexity of the network and the number of training parameters. If the *L* layer is the convolution layer, Equation (13) of the one-dimensional convolution layer is

(13) $$\begin{array}{r} x_{j}^{l}=f\left(\overset{M}{\underset{i=1}{\sum }}x_{i}^{l-1}*k_{ij}^{l}+b_{j}^{l}\right) \end{array}$$

where *k* represents the convolution kernel, *j* represents the number of convolution kernels, and *M* represents the number of channels of the input *x*(*l* − 1) of the upper layer. *b* is the offset of the convolution kernel, and * stands for the convolution operation. *f*(·) is the activation function.

### 4.2. Activation Layer

What is done in the convolution layer of the upper layer is to process the input features in the way of convolution, i.e., to assign a weight to each pixel point. This operation is linear. But for samples, they are non-linear separable instead of linear separable. Thus, we add the activation function Relu Equation (14) here.

(14) $$\begin{array}{r} f\left(x\right)=max\left(0,x\right) \end{array}$$

As a non-linear factor, the activation function added to the model can make the model more expressive and better fit the data.

### 4.3. Pooling Layer

The downsampling stage is after the convolution layer and the number of feature graphs increases. This leads to the expansion of the data dimension, which is not conducive to calculation. Therefore, the average pooling or maximum pooling is used to process each feature map in this stage. The average pool is calculated according to the size of the predetermined pool window, and the maximum pool method selects the maximum parameter within the predetermined window range as the output value. In our study, the maximum pooling operation is adopted. The pooling kernel size is 1*2, the step size is 1, and there is no filling.

### 4.4. Connection Layer

After passing through the convolution layer, the data scale is channel × features, and the feature dimension needs to be straightened into one dimension. At this point, the full connection layer of the node is connected with all neuron nodes output from the feature mapping of the previous layer, and the activation function is softmax function. If the final pooling layer is l+1 and output to the full connection layer, then the output of the full connection layer is Equation (15).

(15) $$\begin{array}{r} h\left(x\right)=f\left(w^{l+1}\cdot x^{l+1}+b^{l+1}\right) \end{array}$$

where *w* represents the weight of each feature, and *b* represents the offset. *F*(·) represents the activation function.

### 4.5. Loss Function

It is used to calculate the error between the classification prediction label and the actual label. The classification cross entropy is used as the loss function, and the probability distribution is compared with the real distribution. *L*_1_, *L*_2_, …, and *L*_*T*_ are represented by the one-hot encoding strategy. The loss function can be calculated as Equation (16).

(16) $$\begin{array}{r} Loss=-\overset{T}{\underset{i=1}{\sum }}\overset{M}{\underset{j=1}{\sum }}L_{i,j}*logp_{i,j} \end{array}$$

where *T* is the number of verification data samples, *M* is the number of classes, *p*_*i,j*_ is the predicted value obtained from the fully connected layer, and *L*_*i,j*_ is the true value.

### 4.6. SparNet

**Figure 4:**
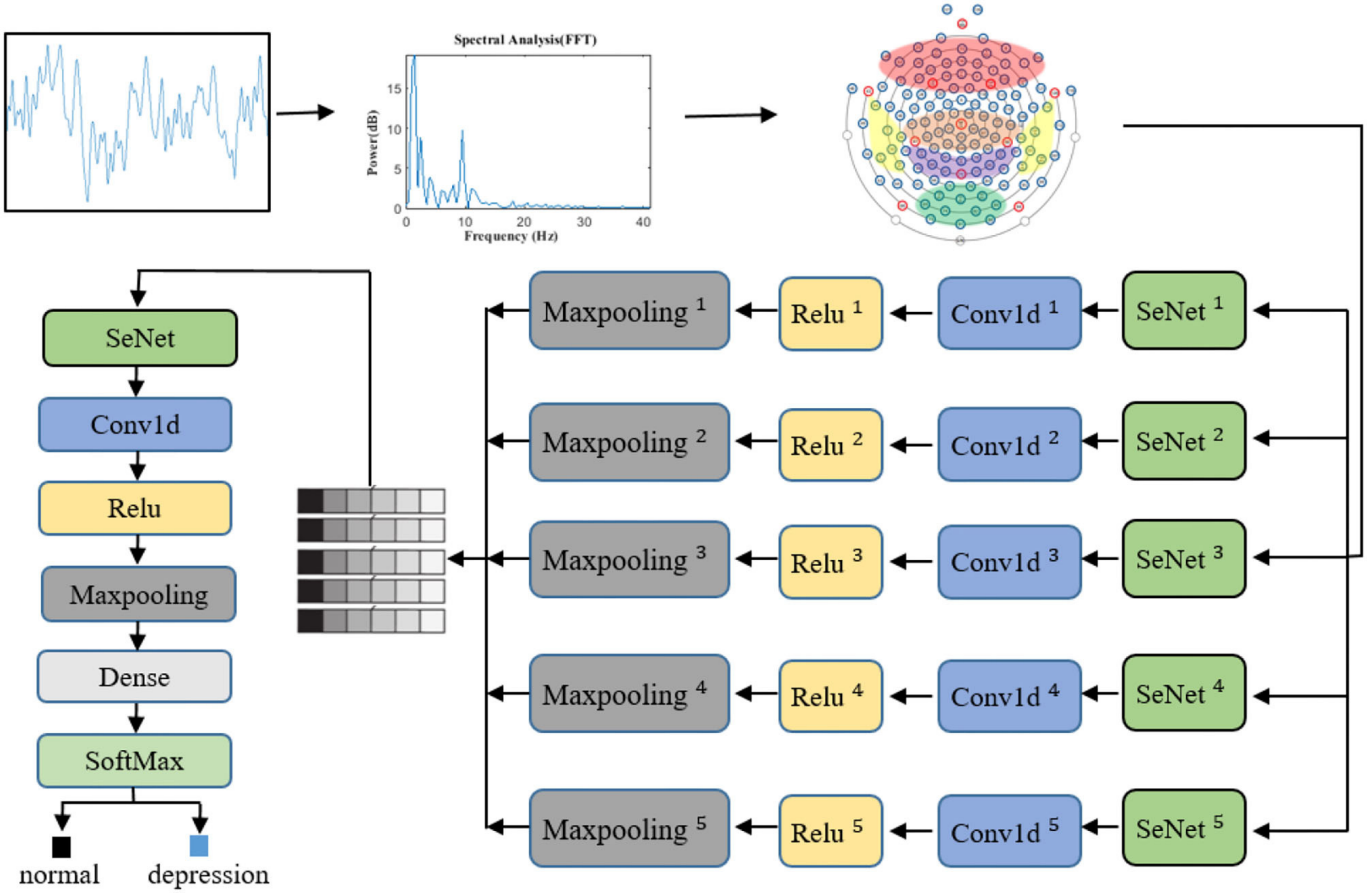
The proposed architecture of the CNN model based on SENet and SparNet. Where ^**^ represents the level of the convolution filter. Conv1d is a one-dimensional convolution operation.

In view of the advantages of CNN, one-dimensional CNN is used to extract the spatial frequency features of EEG signals. In the proposed SparNet network architecture, each layer is directly connected to each other in feedforward mode. In Figure 4, the SparNet network consists of six sub-CNNs. Each sub-CNN consists of a SENet, Conv1d, Relu, and Maxpooling. Five of them are at the same level to form a network of parallel structures to operate the brain region, and the last convolutional operation performs a processing operation on the global brain. Finally, there is a fully connected layer output result. The SENet module is added to each sub-network to increase the attention mechanism between channels and brain regions. Table 1 details each layer of the proposed SparNet network and the parameters of each layer.

**Table 1:** Detailed information about the proposed SparNet deep model.

**No**	**Names of layers**	**Kernel size**	**Parameters of layers**
0	Input	—	—
1	SENet^(1−5)^	—	Reduction = 2
2	Conv1D^(1)^^(3−5)^	12*5	Stride = 1
	Conv1D^(2)^	25*5	Activation = Relu
3	MaxPooling1D^(1−5)^	2	Stride = 2
4	SENet	—	Reduction = 2
5	Conv1D	5*2	Stride = 1, Activation = Relu
6	MaxPooling1D	2	Stride = 2
7	Dense	—	Neurons = 2
8	Softmax	—	—

The ^**^ represents the level of the convolution filter: SENet^(1−5)^ represents the SENet of the 1st to 5th layers.

First of all, the same sample size is kept between the subjects. We use the sliding window 2s non-coincidence method to slice, and the sample size of each subject is 148*2s. In this study, since the recognition features of depression patients are mainly in the frequency domain signals, we manually extracted the features in this step. The EEG signals are transferred from the time domain to the frequency domain (1–40 Hz) by a fast Fourier transform, and the frequency domain signal is used as the input of the neural network. The channels are sorted out before entering the first layer of the model. The channels are divided into five regions according to the brain regions. At this point, the data size of each brain region is channeled *40. Before entering the convolution layer, the SENet module will adjust the weight of each channel, and then the space frequency domain features of each brain region will be extracted through convolution operation and maximum pooling. The features of five brain regions will be spliced to form a feature scale of 5*features, where 5 represents five brain regions. The SENet module then weights each brain region based on global features. After the convolution operation, the output of the full connection layer is entered. Figure 4 shows the whole signal processing process of the network.

### 4.7. Characteristics in the Space Frequency Domain

Peng et al. (2019) and Jiang et al. (2021) showed that the EEG signals of depressed patients had better feature representation in the frequency domain, where absolute power and relative power in the frequency domain were of great help in identifying patients with depression. Moreover, the expression of EEG signals in the power spectrum of patients with depression is obviously different from that of healthy people. Stark et al. (1997) and Lin et al. (2013) showed that in the spatial domain of channels, the brain functional connections of patients with depression were different from those of normal people, and the accuracy of classification of patients with depression was significantly improved.

Therefore, this article aims to combine frequency domain and spatial domain to effectively identify patients with depression in space-frequency domain. Based on the characteristics of the brain, it is divided into five brain regions. First, the local space-frequency domain is explored, and then the feature extraction of the global space-frequency domain is carried out. The brain regions are shown in Table 2.

**Table 2:** Channels in different brain regions are selected.

C	E36-E104,E30-E105,E41-E103,E37-E87,E42-E93,E47-E98
F	E19-E4,E22-E9,E24-E124,E27-E123,E32-E1,E33-E128
O	E70-E83,E71-E76,E69-E89,E74-E82,E73-E83,E75,E81
P	E52-E92,E60-E85,E51-E97,E67-E77,E59-E91,E72,E62
T	E58-E96,E45-E108,E114-E44,E100-E46,E102-E57,E50-E101

**Figure 5:**
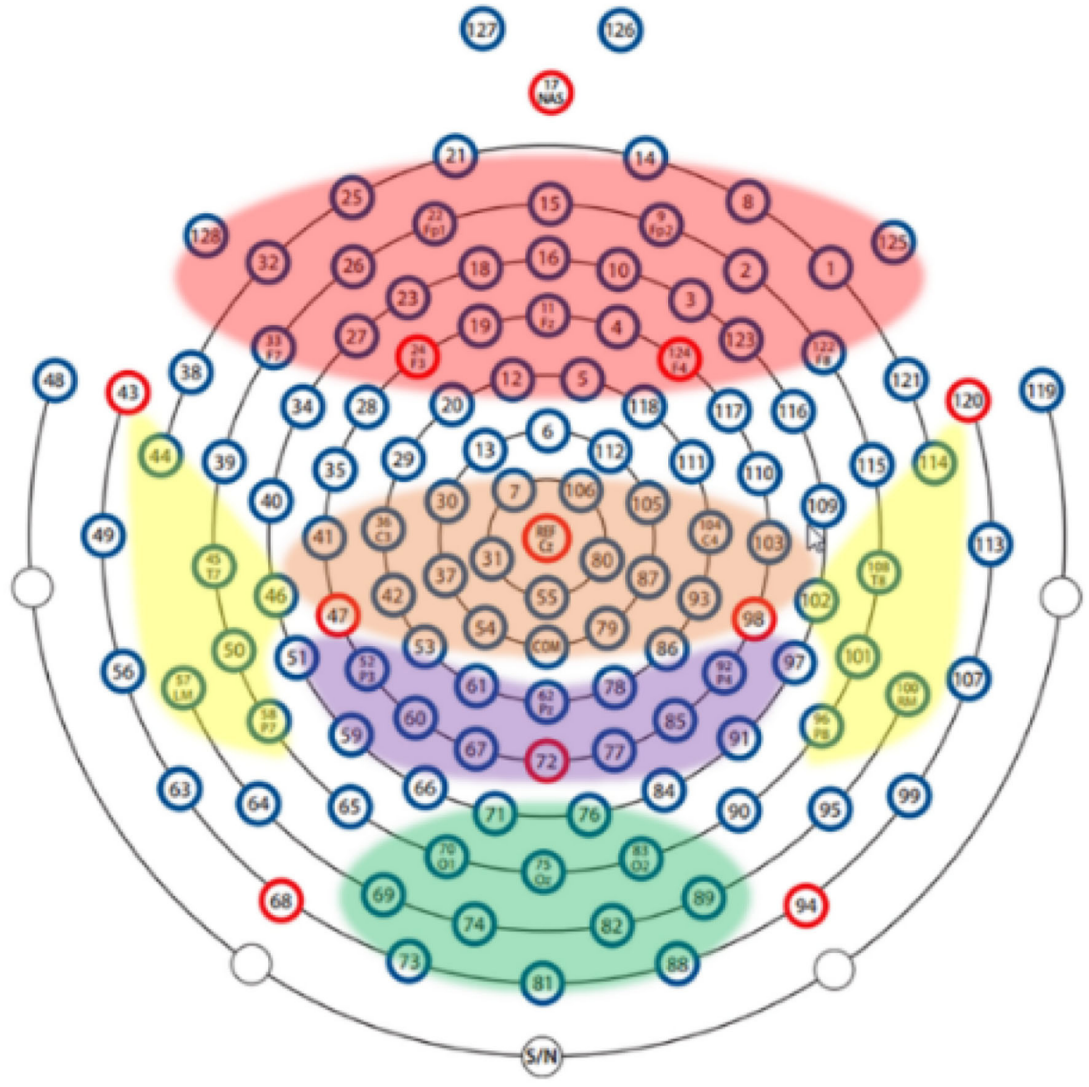
The division of the brain between different brain regions.

In order to explore the influence of various brain regions on patients with depression, we divide the brain into five parts as shown in Figure 5: central region, frontal lobe region, occipital lobe region, parietal lobe region, and temporal lobe region. The first layer of the SparNet network has five parallel sub-CNNs corresponding to the five brain regions. Due to the tightness between brain regions, we initially select 12 channels in each brain region to carry out space frequency domain characteristics of brain regions. In order to accurately locate the channels in each brain region, we obtained the channel location information from Luu and Ferree (2005) by referring to the 128-channel HydroCel Geodesic Sensor Net device. Each channel contains spatial information about the brain. Additionally, in order to avoid the influence of multiple brain regions on a single channel, we select 12 channels distributed in the central location of each brain region without selecting channels at the edge of the brain region. The channels are then added individually according to each brain region's contribution to the recognition of depression. The selection of preliminary channels is shown in Table 2.

## 5. Results

### 5.1. Evaluation

There are 48 subjects in total. In order to better generalize the model, we adopt dataset division among subjects. A total of 24 sets are obtained by combining a depressed patient with healthy control, using the one-subject cross-validation (LOSOCV) to assess the generalization ability of each classification model.

In this study, accuracy, sensitivity, and accuracy based on the confusion matrix are used as the performance evaluation indexes. Sensitivity (recall rate) is defined as the percentage of patients with MDD predicted in all MDD patients (TP+ FN), and precision is defined as the percentage of healthy controls predicted in all healthy controls (TP+FP). Accuracy is defined as the percentage of correctly classified patients with MDD and healthy controls. F1 index takes into account both model accuracy and recall rate and is defined as the harmonic mean of model accuracy and recall rate.

(17) $$\begin{array}{r} Accurary=\frac{TP+TN}{TP+TN+FP+FN} \end{array}$$

(18) $$\begin{array}{r} Sensitivity=\frac{TP}{TP+FN} \end{array}$$

(19) $$\begin{array}{r} Precision=\frac{TP}{TP+FP} \end{array}$$

(20) $$\begin{array}{r} F_{1}Score=2\times \frac{Precision\times Sensitivity}{Precision\times Sensitivity} \end{array}$$

The Receiver Operating characteristic (ROC) curve is used for evaluation. The ROC curve is widely used in binary classification evaluation, which evaluates sensitivity and specificity against several thresholds.

### 5.2. Partial Results

**Figure 6:**
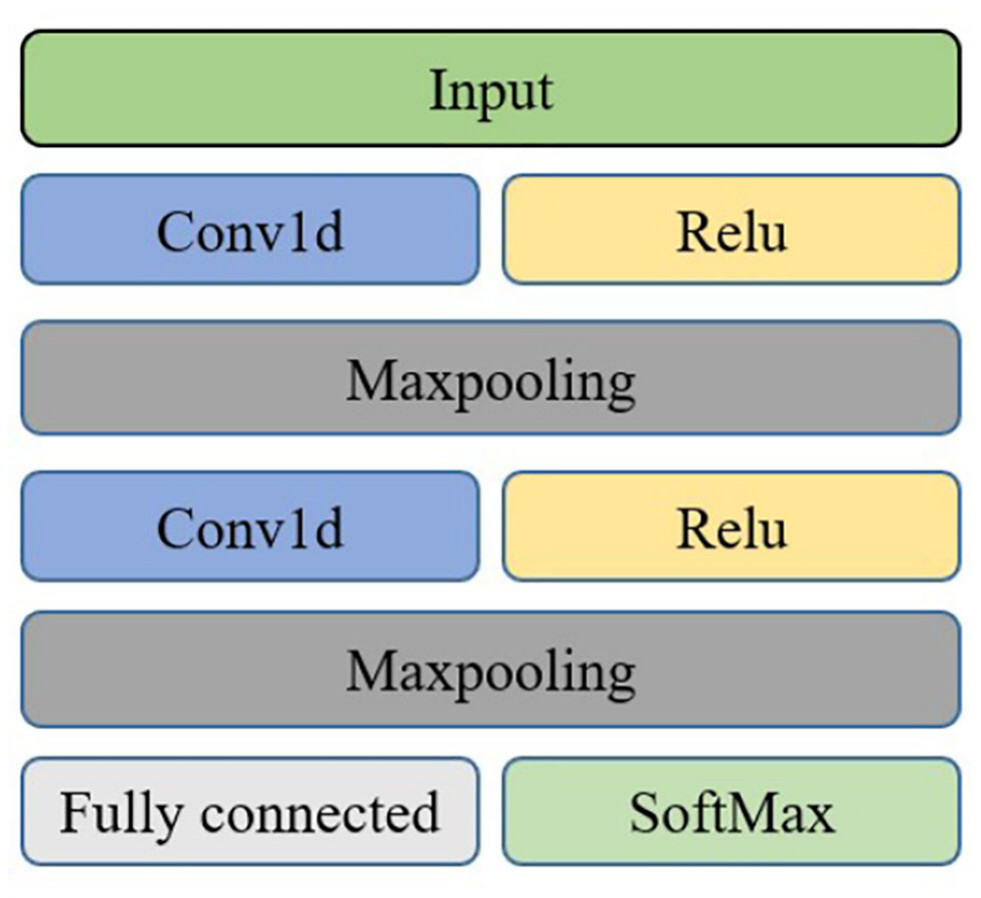
Network structure diagram of brain regions.

The brain is divided into five regions: frontal, central, parietal, occipital, and temporal. In order to more effectively identify people with depression, we explored the importance of different brain regions. The importance of 12 channels in each brain region is evaluated. 1DCNN is performed on each of the five brain regions, and the evaluation index is the average accuracy after each fold. The neural network model of brain regions is shown in Figure 6.

**Figure 7:**
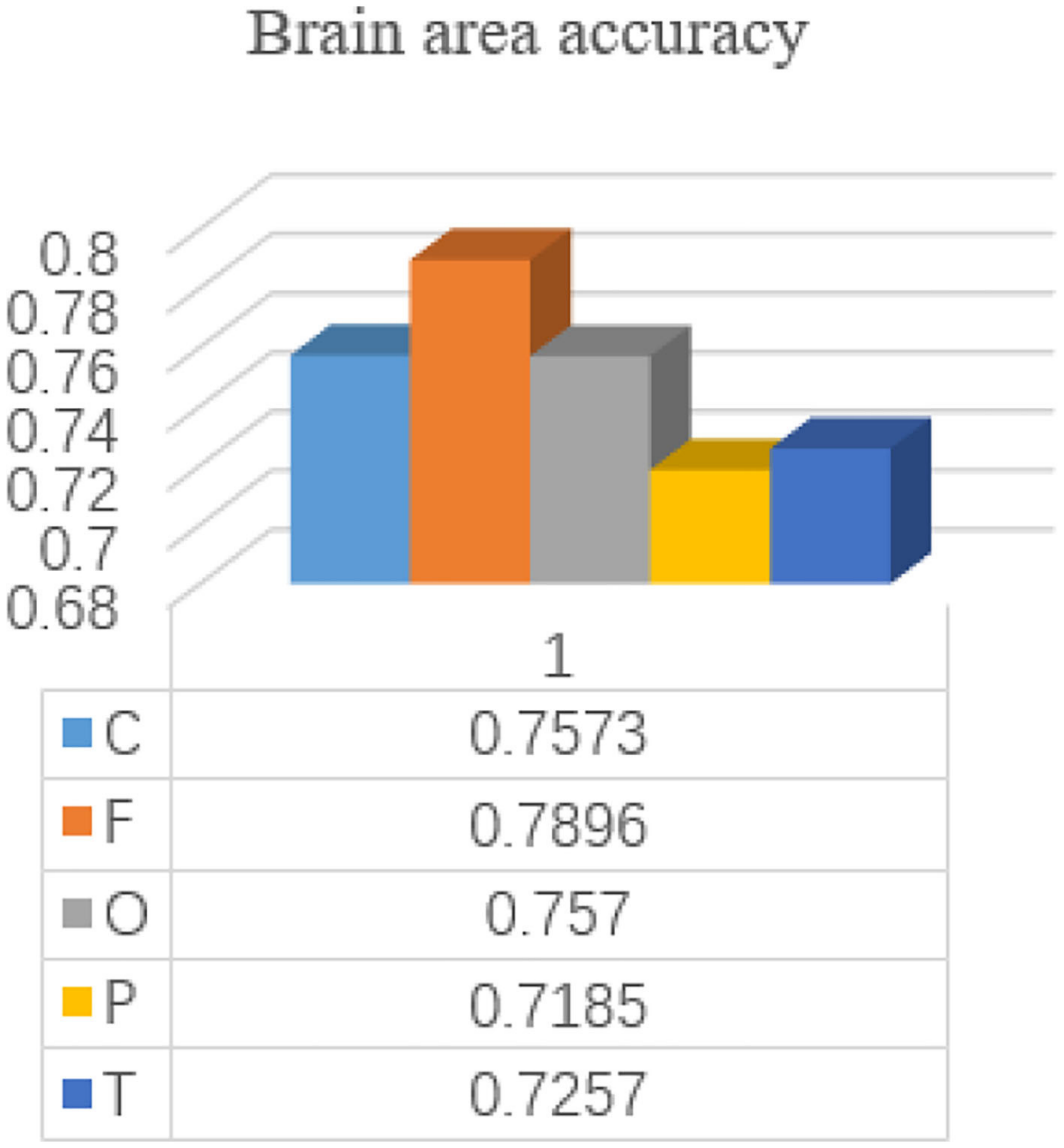
The accuracy of different brain regions in identifying people with depression.

Each subject is included in the test set by a 24-time retention cross-validation method for each brain region. The accuracy of each brain region is shown in Figure 7.

As can be seen from the results, the frontal lobe has a greater contribution to the identification of patients with depression compared with other brain regions, which is consistent with the results of Jiang et al. (2021), indicating the feasibility of the frontal lobe channel detection for depression. Wan et al. (2020) also prefer the frontal lobe channel in terms of channel selection. Depression patients with low moods will lead to emotional differences from normal people. Since the frontal lobe is the main brain area for emotional processing, the frontal lobe is more important for the identification of depression patients. Thus, in order to better explore the characteristics of the global space frequency domain, the number of channels in the frontal lobe is increased to 25 channels, E19-E4, E22-E9, E24-E124, E27-E123, E32-E1, E33-E128, E15, E11, E1 - E32, E27 - E123, E13 - E112, E29 - E111, E28-117, the E6, E121 - E38, E34 - E36, combined with the cerebral cortex region occupied by the frontal lobe.

### 5.3. Global Results

The software Matlab 2020b is used to preprocess the data, and the EEG data are segmented with a 2s window length. After that, the time domain signal is converted to the frequency domain as the characteristic input of the model. The space frequency domain features of the EEG signal will be extracted by a two-layer convolution operation. In addition, in the convolution operation, each channel will be adjusted according to the parameters of a convolution kernel, and will not be affected by the parameters of other channels. Therefore, the channel grouping order has no significant impact on performance.

**Figure 8:**
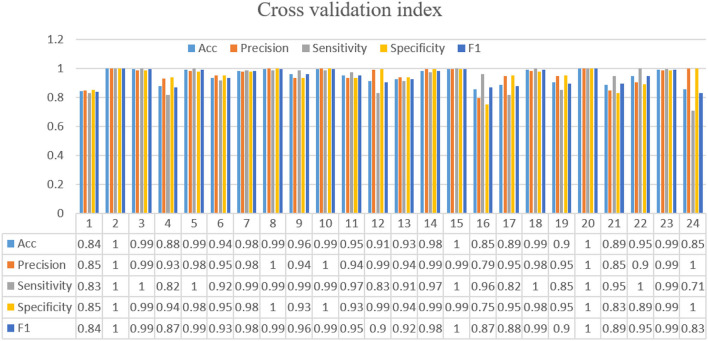
Twenty-four-fold cross-validation results including accuracy, precision, sensitivity, specificity, and F1 evaluation indicators.

**Figure 9:**
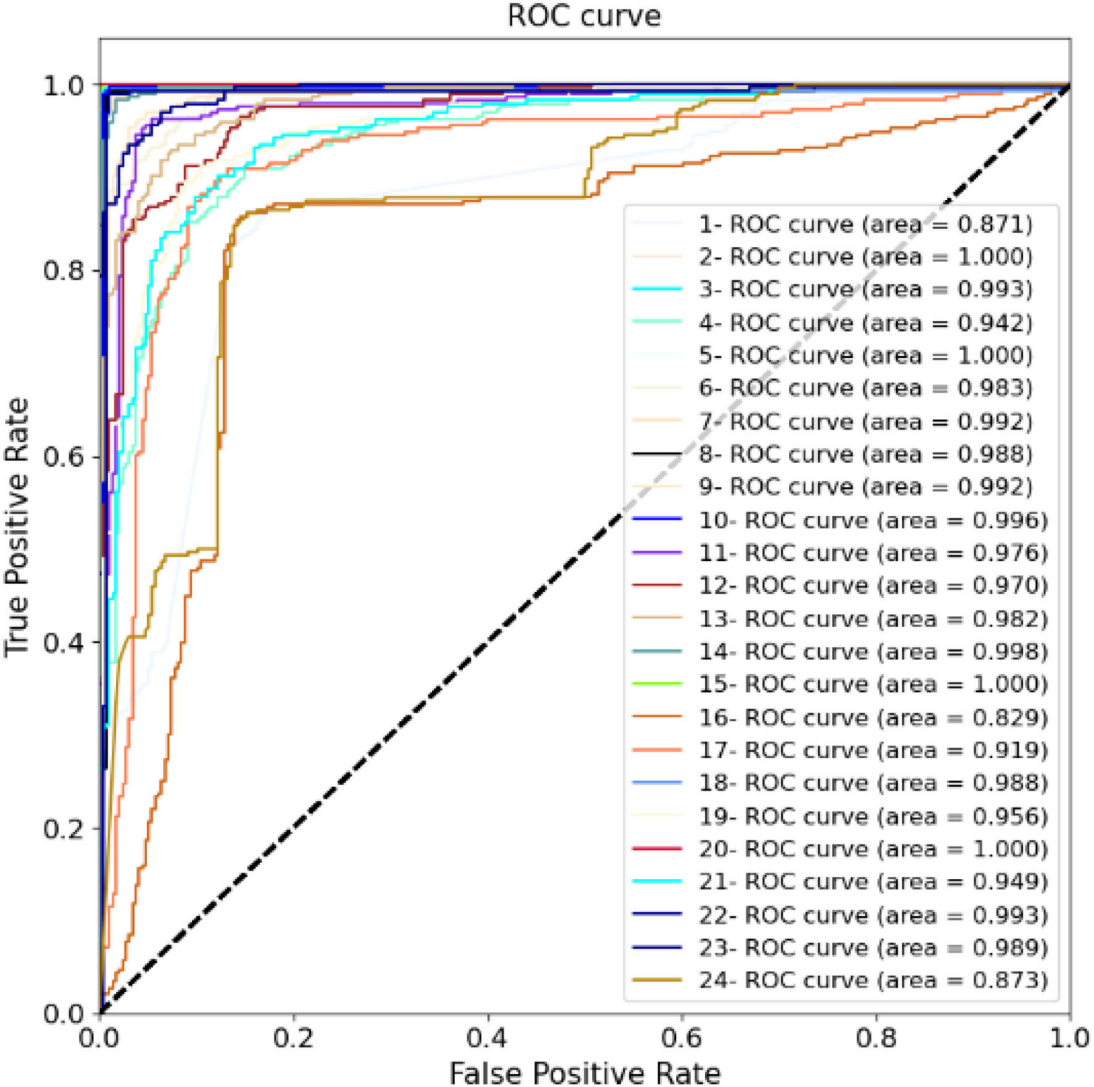
Twenty-four-fold cross-verified ROC curves and areas.

For training the model, the batch size is 8, and each network is trained with 50 epochs. The cross entropy is selected as the loss function. In the optimization stage, the RMS algorithm is selected to obtain better results and a shorter running time. If the loss function is verified not to improve after 10 consecutive epochs, the early stop criterion is used. Figure 8 shows the calculation results for each fold. The ROC and AUC values of the above methods are shown in Figure 9 for each fold. Due to the differences between the subjects, the minimum AUC area obtained is 0.829, and there are 16-fold AUC areas over 0.95, which also demonstrates the effectiveness of the model. The results of cross-validation are statistically analyzed. The mean values of F1, Acc, Precision, Sensitivity, Specificity are 0.947, 0.953, 0.937, 0.951, and 0.942, respectively. The standard deviation of F1, Acc, Precision, Sensitivity, Specificity are 0.054, 0.056, 0.082, 0.063, 0.056, respectively.

**Figure 10:**
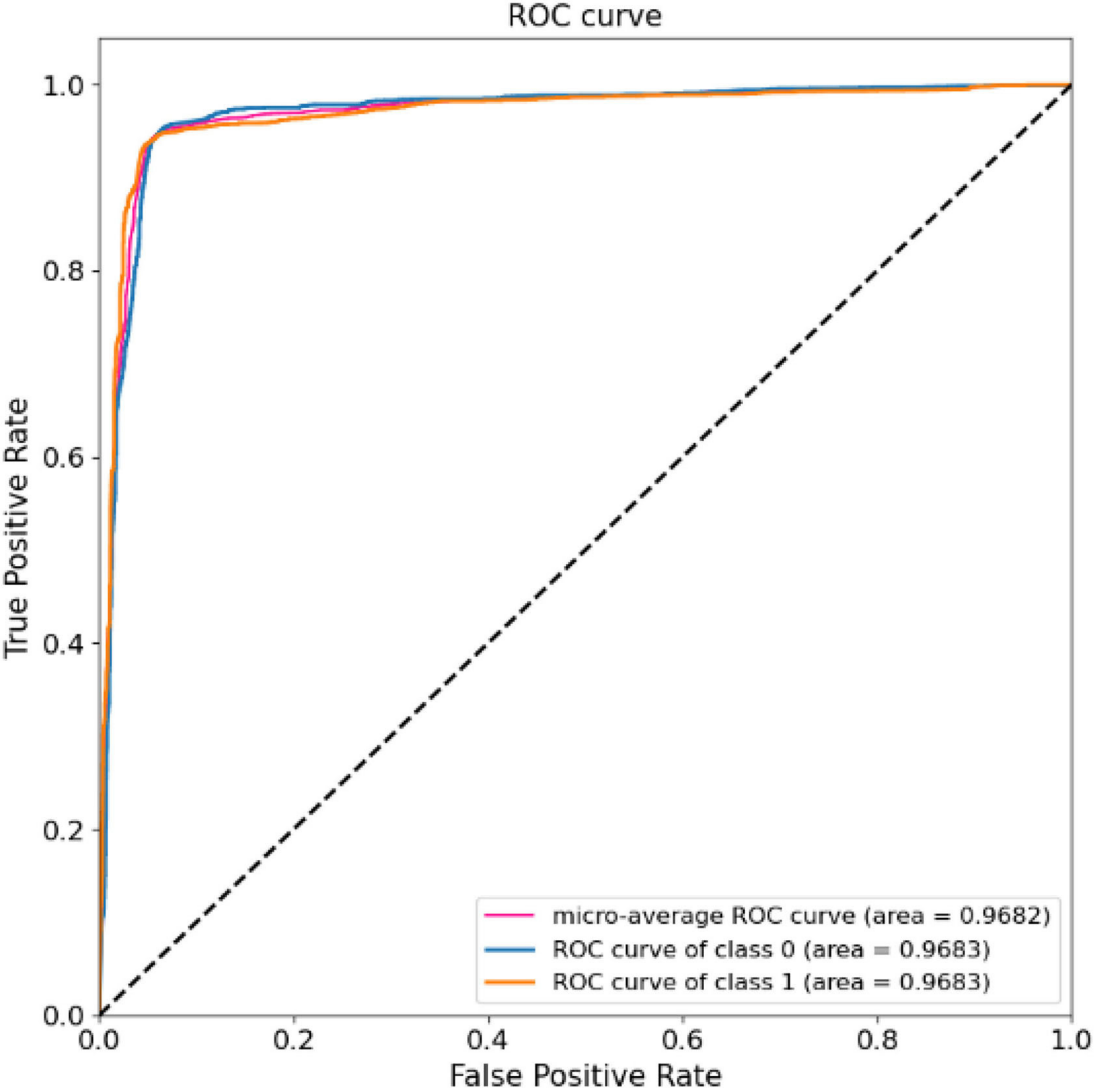
Receiver Operating characteristic (ROC) curves and area of overall EEG data tests.

The results of 24-folds are combined and analyzed, and the evaluation indexes obtained are shown in Table 3, in which the accuracy rate reaches 94.37%. At the same time, the ROC and AUC values of the above methods are shown for the overall results, including the ROC and AUC values of the individual categories of normal people and depressed patients, as shown in Figure 10. The AUC area of the overall data is 0.9682. All the evaluation indicators decrease when the input characteristics in SparNet are time domain signals. The results are shown in Table 4, named “With Time Domain”. Thus, the frequency domain feature is more effective than the time domain feature.

**Table 3:** Performance values obtained when testing the SparNet model using EEG data.

**Class**		**Predicted depression**	**Normal**	**Acc**	**Precision**	**Sensitivity**	**Specificity**	**F1**
Actual	Depression	3,327	225	0.9437	0.9375	0.9507	0.9366	0.9440
	Normal	175	3,377		0.9500	0.9366	0.9507	0.9432

**Table 4:** Comparison of results before and after adding SENet module.

	**Acc**	**Precision**	**Sensitivity**	**Specificity**	**F1**
SparNet	0.9437	0.9375	0.9507	0.9366	0.9440
With time domain	0.8851	0.9189	0.8607	0.9104	0.9130
Without the SENet	0.9124	0.8953	0.9341	0.8907	0.9143
Without smooth	0.9037	0.9099	0.9042	0.9104	0.9070

For the proposed model, in order to verify the effectiveness of the attention mechanism in identifying patients with depression, six SENet modules are removed from the model and the comparison before and after the results are shown in Table 4.

It can be seen from the results that adding an attention mechanism into the model can improve the learning ability of the model. The accuracy rate of depression identification is improved by 3.13%, and all the evaluation indicators are also improved.

In this article, after converting the EEG signals into the frequency domain, the smoothing operation is performed on the frequency domain features. The comparative experiments are conducted before and after feature smoothing, and the experimental results are shown in Table 4.

It can be seen from the results that the accuracy is improved by 4 % using the smoothed features as the input. Furthermore, we can observe that all the evaluation indicators are improved at the same time. The calculation cost of the network is calculated. The parameter number of the SparNet network is 8,725, and the network parameter without the SENet module is 455. The number of network parameters without smooth is 8,725, mainly to illustrate the necessity of feature smoothing for neural networks. The SparNet parameters are mainly contributed by the SENet module.

## 6. Discussion

Screening for depression is very important for early diagnosis and treatment. However, the previous diagnosis of depression is confined to a manual questionnaire survey and feature extraction, which is subject to many limitations. For example, a questionnaire survey required experienced doctors, while feature extraction required a lot of manpower to find the characteristics of relevant indicators. The deep learning methods can overcome this limitation and can be used anywhere without highly trained experts. In this study, we use feature smoothing and deep learning for the automatic detection of patients with MDD and healthy controls with good performance. The attention mechanism is combined with 1DCNN, and the spatial-frequency domain features are extracted by brain regions. The accuracy rate of our model reaches 94.37%.

The main innovation of this study is to make full use of the spatial and frequency domain characteristics of the brain. We also try to smooth the input of the neural network. Experimental results show that the frequency domain characteristics of the input smoothing processing can effectively improve the identification accuracy of patients. In addition, due to the characteristics of the convolutional neural network, the brain is divided into different regions for feature extraction of parallel structures.

It can be seen from the final results that adding the SE module into the 1DCNN neural network can make the model have higher accuracy. This is because the convolutional layer provides a powerful feature fusion technology, although the weights between channels are unified by default. The SE module can better highlight the importance of different channels. At the same time, although there has been a feature of converting the EEG signal into the frequency domain as the input, the segmentation of brain regions according to the brain's structure can make the spatial information of brain regions better interpreted. The results for each brain region and the final results for the whole brain region show that although each brain region is helpful in identifying depression, the characteristics of the whole brain are better. It could be thought that a large amount of space-frequency information is lost for a single brain region and cannot get a significant effect.

Table 5 is a comparison of results on the same data set. It can be seen that the accuracy of this study is higher than that of traditional machine learning methods for linear and non-linear extraction. Additionally, compared with Peng et al. (2019), we use fewer channels to achieve better accuracy and obtain the best results in this dataset in automated detection of patients with depression and healthy controls, which could provide better solutions for future clinical applications. Compared with Zhang et al. (2020), the combination of multi-parallel 1DCNN and SE modules in this study tests the importance of space-frequency information and other advantages. However, the main shortcoming of this study is the data size to train the network, which can be overcome by simplifying the deep model. Our future goals are to further expand the experimental space, collect more samples, and apply the developed methods to other types of EEG data.

**Table 5:** The confounding matrix and evaluation index are used to compare the classification results of SparNet and correlation methods in the same data set.

**References**	**Methods**	**Channels number**	**Classification methods**	**Accuracy (%)**
(Sun et al., 2020b)	Linear features, non-linear features, PLI	16 Channels	ReliefF, LR	82.31
(Peng et al., 2019)	PLI	128 Channels	Kendall rank correlation coefficient+SVM	92.73
(Sun et al., 2020a)	Linear features, non-linear features, PLI, network measures	128 Channels	C4.5, BFDT, LR	84.18
(Zhang et al., 2020)	Time domain feature	3 Channels	1DCNN	75.29
SparNet	Frequency domain feature	73 Channels	1DCNN	**94.37**

*The bold value is the accuracy derived from the SparNet*.

## 7. Conclusion

The main study in this article is using the novel neural network called SparNet based on the EEG signals to identify depressed people or not. First, the denoising method of the phase space reconstruction is used to denoise and clean the data. Second, the input features are smoothed before the frequency domain features are input into the model. Third, a new model called SparNet is proposed to extract the space-frequency domain features of the local brain regions and the whole-brain. Finally, the cooperating of the attentional mechanisms to the model improves the identification accuracy of the patients with depression. Compared with other methods, the proposed model can obtain a better classification performance. From the results of the local brain regions, it can be seen that the frontal lobe plays a better role in the identification of patients with depression. From the results of the global brain region, it can be seen that the combination of the spatial features and the frequency domain features can effectively improve the accuracy of depression identification. The combination of features of the different brain regions may be the focus of future research. The methods and findings of this study may contribute to the wider application of the diagnosis of deep depression in clinical applications and neuroscience research.

## Data Availability Statement

The original contributions presented in the study are included in the article/supplementary material, further inquiries can be directed to the corresponding author/s.

## Author Contributions

XD and XF: conceptualization and writing—review and editing. XD, XF, and XL: methodology. XF: software, data curation, and writing—original draft preparation. XD, XF, XL, and KS: validation and investigation. All authors have read and agreed to the published version of the manuscript.

## Funding

This study was supported in part by the Natural Science Foundation of Chongqing under Grant cstc2020jcyj-msxmX0284, in part by the Scientific and Technological Research Program of Chongqing Municipal Education Commission under Grant KJQN202000625, in part by the National Natural Science Foundation of China under Grants 61806033, 61703065, and 62101084, and in part by the Key Industry Core Technology Innovation Project of CQ under Grant cstc2017zdcy-zdyfX0012.

## Conflict of Interest

The authors declare that the research was conducted in the absence of any commercial or financial relationships that could be construed as a potential conflict of interest.

## Publisher's Note

All claims expressed in this article are solely those of the authors and do not necessarily represent those of their affiliated organizations, or those of the publisher, the editors and the reviewers. Any product that may be evaluated in this article, or claim that may be made by its manufacturer, is not guaranteed or endorsed by the publisher.
